# Graphene and Graphene-Based Nanomaterials for DNA Detection: A Review

**DOI:** 10.3390/molecules23082050

**Published:** 2018-08-16

**Authors:** Xin Wu, Fengwen Mu, Yinghui Wang, Haiyan Zhao

**Affiliations:** 1 George S. Ansell Department of Metallurgical and Materials Engineering, Colorado School of Mines, Golden, CO 80401, USA; 2 State Key Laboratory of Tribology, Department of Mechanical Engineering, Tsinghua University, Beijing 100084, China; hyzhao@tsinghua.edu.cn; 3 Department of Precision Engineering, The University of Tokyo, Tokyo 113-8656, Japan; 4 Kunshan Branch, Institute of Microelectronics, Chinese Academy of Sciences, Suzhou 215347, China; wangyinghui@ime.ac.cn

**Keywords:** DNA detection, graphene, graphene-based nanomaterials, biosensors

## Abstract

DNA detection with high sensitivity and specificity has tremendous potential as molecular diagnostic agents. Graphene and graphene-based nanomaterials, such as graphene nanopore, graphene nanoribbon, graphene oxide, and reduced graphene oxide, graphene-nanoparticle composites, were demonstrated to have unique properties, which have attracted increasing interest towards the application of DNA detection with improved performance. This article comprehensively reviews the most recent trends in DNA detection based on graphene and graphene-related nanomaterials. Based on the current understanding, this review attempts to identify the future directions in which the field is likely to thrive, and stimulate more significant research in this subject.

## 1. Introduction

DNA is the molecule that encodes genetic instructions. As the blueprint of life, DNA detection has received significant attention in recent years due to its promising applications in disease diagnosis and treatment, forensic analysis, food safety evaluation, environmental monitoring, and so on. There are two significant challenges in DNA detection, one is how to perform rapid and robust detection of rare DNA amongst a huge amount of background DNAs or other biomolecules, the other is how to accurately and rapidly sequence the DNA, which was also the target set by the $1000 genome project [1]. There are a lot of techniques proposed to detect DNA, which can be categorized into the label based and label-free based methods. The labelling of the target DNA with dyes or enzymes could significantly enhance the sensitivity, while at the expense of simplicity and sample preparation [2]. As a contrast, the label-free detection methods provide the advantages such as ease of miniaturization, inexpensive device fabrication, operation simplicity and rapid detection [3]. Based on the signal that the device detected during the interaction between different DNAs and the material surface, the DNA sensors can also be divided into electrochemical [4,5,6], electronic [7] and optical [8] biosensors. With the requirement of high sensitivity and high specificity, micro- and nanotechnologies have shown emerging potential in DNA diagnostics. Nanomaterials have the capability to offer improved biocompatibility, additional binding sites and higher signal intensities (via enhanced electrical properties) compared with traditional materials in DNA sensors [9,10,11,12]. Among the nanomaterials used in DNA detection, graphene is receiving increasing attentions these years.

**Figure 1:**
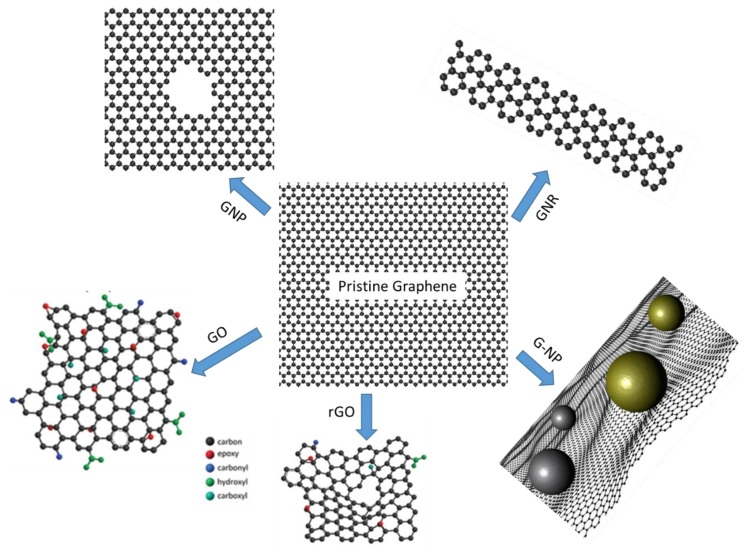
Graphene and graphene-based nanomaterials for DNA detection.

Graphene, as a two-dimensional (2D) material, is constituted by only one-layer sp2-bonded carbon atoms with a honeycomb structure. Due to its extraordinary properties in electrical, optical, mechanical, chemical aspects [13,14,15,16,17,18,19,20], and great application potentials in flexible electronics, solar cells, supercapacitors, optoelectronic devices and biosensors, etc. [21,22,23,24,25], graphene is becoming the research hotspot in recent years. Especially in the field of DNA detection, the application of graphene could highly improve the sensitivity and selectivity of the testing technique. For example, the ultrathin thickness of graphene [26] could make the sequencing of DNA molecule at a single nucleobase resolution be possible. Besides, the large surface area (up to 2630 m^2^/g) [27] provides enough sites for DNA anchoring, which leads to the efficient and rapid detection of DNA. Also, the high charge carrier concentration and mobility, low charge-transfer resistance, good biocompatibility and easy biological and chemical functionalization [28], demonstrate graphene to be a promising material in the development of rapid, accurate, sensitive and portable DNA detection techniques. However, the graphene itself is usually hard to be directly applied in the DNA detection due to some of the intrinsic drawbacks, like the hydrophobicity, zero band gap, chemical stability. It’s usually necessary to process graphene into nanostructures, such as the graphene nanopore (GNP), graphene nanoribbon (GNR), graphene oxide (GO), reduced graphene oxide (rGO), and graphene-nanoparticle (G-NP) hybrid materials, as shown in Figure 1. There are a lot of reports demonstrating the adaptation of graphene and graphene based nanomaterials for the applications of DNA detection. For example, as a label-free and amplification-free single-molecule approach, the GNP structure is outstanding in the application of DNA sequencing [29,30,31,32], similar as the graphene nanogaps [33]. Meanwhile, pristine graphene and functionalized graphene (GO, rGO) present strong capability in the synthesis of electrochemical, electronic and optical biosensors for DNA detection [34,35,36,37,38]. The graphene-nanoparticle hybrid structures could display the individual properties of nanoparticles and graphene. What’s more, they exhibit additional advantageous and synergistic properties as well, which augments their potentials for DNA detection [39,40,41].

The research advancements of DNA detection based on graphene and graphene-related nanomaterials could stimulate further research interest to realize and expand the bioapplications of graphene-based nanomaterials. In this perspective, by aiming at speeding up the realization of quick and accurate DNA detections in the market, we reviewed recent progresses and achievements in the topic of DNA detection using graphene and graphene-based nanomaterials. This paper could also offer useful information for the beginners in this area.

## 2. GNPs, GNRs and Graphene Nanogaps Based DNA Sequencing

**Figure 2:**
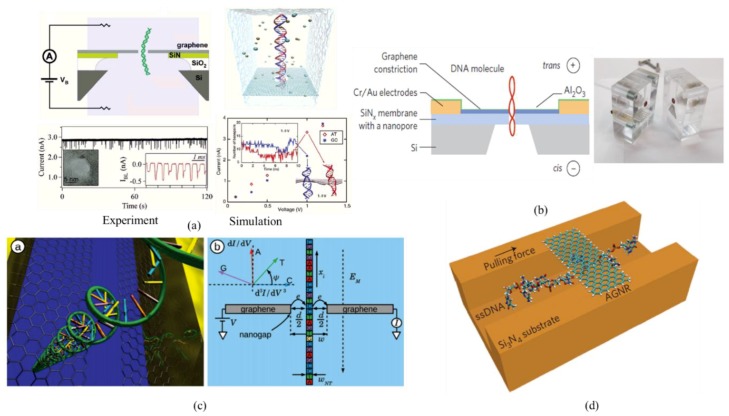
Graphene based nanostructures for DNA sequencing. (**a**) GNP based DNA sequencing. Illustrations of experimental [45] and simulation studies [49] are shown respectively; (**b**) Schematics of the GNR transistor-nanopore measuring set-up and photograph of the fluidic cell [69]; (**c**) Schematic representation of the translocation of DNA molecule through a graphene nanogap [75]; (**d**) A nanochannel device with an armchair GNR through which a ssDNA passes [86].

DNA sequencing is important in a lot of scientific fields, including genetics, biomedical science, clinical diagnostics, molecular biology, anthropology and forensic sciences [42]. Nanopore based DNA sequencers were proposed as the fourth-generation DNA sequencing technology, which shows the potential to sequence the entire human genome quickly and reliably for less than $1000 [43,44]. The basic concept of nanopore DNA sequencing is based on the modulation of the signal in a specific and measurable way as the nucleic acid passes through the pore. In order to achieve the high sensitivity, accuracy, and speed of DNA sequencing, the nanopore materials should be thermally and mechanically stable and chemically robust, with low background noise and high sensitive to signal change. Especially the thickness should be comparable to the spatial interval between neighboring DNA nucleotide. Graphene, emerging as a new 2D material in recent years, is constituted by single-layer carbon atoms with sp2 hybridization. With exceptional properties, graphene was demonstrated to be outstanding among the nanopore materials to be used in DNA sequencing. There are many experimental and simulation studies talking about the feasibility, mechanism, and influence factors relating to the GNP based DNA sequencing. In 2010, three different groups proposed to use the GNP for DNA sensing and experimentally demonstrated the specific current blockade due to the DNA translocation events [45,46,47], which indicates the promising future for GNP based DNA sequencing. Figure 2a left gives the typical experiment setup for this approach and the ionic current signal during the translocation events. The device is mainly made up of three parts: (1) Few-layer GNP structure over a silicon nitride (SiN) membrane. The SiN membrane is suspended over a silicon chip with SiO_2_ layer; (2) Microfluidic channels that form reservoirs in contact with each side of the chip; (3) An external circuit to apply the bias voltage and record the current signal. Based on this setup, a larger ionic current blockage by DNA translocation through GNPs was obtained than using the SiN nanopores of the same diameter. Distinct signal can be obtained when the double-stranded DNA (dsDNA) molecule translocate through the nanopore in different configurations (nonfolded, partially folded and fully folded). Though they proved the capability of GNP based nano-devices for the DNA detection, it is still unknown whether the GNP can be used for the DNA sequencing in a single base resolution or not. The atomic simulation approaches were well developed for uncovering the underlying mechanisms of the nanostructures. Then ab initio density functional theory (DFT) [48] and molecular dynamics (MD) simulation [49] were adopted to demonstrate the feasibility of DNA sequencing with ionic current detection through GNPs. Figure 2a right shows the representative MD model for the GNP based DNA sequencing, and the distinct current signal of poly(AT) and poly(GC) under applied voltages. The MD model was built according to the experiment conditions, which means it is also constituted with three parts: the NPG structure, the two reservoirs with KCl solution, and the externally applied voltage. The simulation results demonstrated that the poly(AT) and poly(GC) can be distinguished using the GNP structure at a bias voltage of 1 V. Then in the following several years, a lot of experimental and simulation work was conducted to, on one hand, further uncover how the dominated parameters control the translocation process of the DNA, on the other hand, address the underlying challenges for the realization of this sequencing technology. It was pointed out that the conformational fluctuations of the nucleotides happen easily in the nanopores and the ionic current blockades are highly sensitive to the orientation of the nucleotides [49,50]. In order to achieve the base sequencing, it is essential to control the conformation of DNA nucleotides in the nanopore. Interestingly, the conformational fluctuations of the nucleotides in the nanopores could be significantly reduced by the hydrophobic interactions with the graphene. Because the confirmation of DNAs depends on the external voltage, the physical and chemical properties of the pore, it was proposed to control the DNA confirmation through precise engineering of the nanopore surface. The nanopore geometry is one of the key factors influencing the feasibility of DNA sequencing. In 2013, Garaj et al. [51] experimentally investigated the size effects of the GNP and found some critical sizes of the nanopore for the DNA translocation. In nanopores greater than ~4 nm, dsDNA molecules translocate through the pore either as extended linear molecules or as a folded molecule, while in nanopores with diameters smaller than 3.2 nm, only unfolded molecules were observed. By using theoretical method, Liang et al. [52] demonstrated that the resolution of DNA sequence detection can be improved when the diameter of the nanopore is reduced. Zhang et al. [53] also indicated that to sequence DNA by atomic force microscopy or optical tweezers, the GNP surface should be modified as symmetrically as possible. Besides, the adsorption of DNA onto graphene could result in the unexpected errors, even failure of the test. The DNA molecule may clog the nanopore by strongly sticking onto the membrane. By using AFM mapping of a graphitic surface exposed to the solution containing single-stranded DNA (ssDNA), Schneider et al. [54] confirmed the strong adsorption of DNA on the surface. To minimize this interaction, several methods were proposed, like the deposition of hydrophilic TiO_2_ [45], increasing the pH of the solution [51], non-covalently functionalization [54]. There are also some other factors impacting the efficiency and accuracy of the DNA detection by GNPs, like the number of graphene layers [55,56], mechanical stability of the graphene [49,54], salt concentration and applied bias voltage [49,50,52]. For more information, please refer to some of the recent review papers [32,57,58,59]. Given the fast translocation velocity, the conformational fluctuations, the stochastic translocation of the DNA molecules and the high ionic current noise levels, there are still significant challenges to reach single-base resolution for the nanopore based DNA sequencing. Based on the intrinsic conductivity of graphene, the researchers are trying to find out alternative read-out schemes to replace the blocked current detection. Under this motivation, the GNRs and graphene nanogaps based DNA detection methods are then proposed.

Graphene was proved to be a gapless semiconductor [60], while it can present semiconducting or metallic properties when it’s structured into a nanometer sized ribbon with different chirality [61]. The high in-plane carrier transport rate and its sensitivity to disturbance make GNRs promising platforms for DNA sequencing. There are different types of GNRs-based platforms, among which the GNR with a nanopore structure is mostly investigated. The idea is to measure the transverse (i.e., perpendicular to the DNA molecule) current through a GNR with a nanopore. When DNA bases pass through the nanopore, there are base-specific modulations in the electronic current through graphene nanostructure, enabling measurement of the DNA sequence. The probing of DNA translocations with in-plane current signals in GNR was first demonstrated by Nelson et al. using DFT method [48]. They analyzed the conductance spectra and charge densities in the presence of different nucleobases in the graphene nanopore, and proved this device could discriminate between the four different bases. Meanwhile, the nucleotide conductance spectrum is affected little by its orientation inside the nanopore. Many theoretical and computational studies on transport in GNRs were then conducted to solve the existing difficulties in DNA sequencing by this technique [62,63,64,65,66,67,68]. These studies show that the non-electrostatic base-specific interactions between the DNA bases and the GNR result in alterations of the local density of states around the nanopore, leading to the resistive changes of the nanoribbon that can be measured as the in-plane current running through the ribbon. The first experimental results on DNA translocation through GNR with a nanopore were reported in 2013 by Traversi et al. [69]. In this work, they integrated the solid-state nanopore with a graphene nanoribbon transistor. Figure 2b depicts the schematic of the set-up of this device, and photograph of the fluidic cell. The fabrication of this device started from the creation of a 20 nm thick SiNx membrane, followed by the transfer of a chemical vapour deposition (CVD) monolayer graphene. GNRs were defined using electron-beam lithography (EBL) and oxygen reactive ion etching. Next, electrical contacts were fabricated by EBL, electron-beam evaporation of a Cr (5 nm)/Au (50 nm) metal double layer and lift-off, and a layer of 5 nm Al_2_O_3_ was deposited to minimize the ionic cross-conductance. The nanopore was drilled by a transmission electron microscope (TEM) working in scanning mode. The chips were placed in a custom-made microfluidic chamber to carry out the simultaneous measurements of the ionic current and in-plane current. They found that as the DNA molecules move through the pore, the device can simultaneously measure drops in ionic current and changes in local voltage in the transistor, both of which can be used to detect the molecules. Later on, by using an arrangement similar to Traversi’s experimental setup, Rocha et al. [70] computationally modelled the capacitative current signal and explained the underlying detection mechanism. Similar approach was also reported with wider GNRs [71]. Importantly, the current signals measured with these systems were shown to originate in a capacitive coupling of the potential change at the nanopore that occurs during DNA translocation. Though the capacitive signal reveals the local presence of DNA in the nanopore, it couldn’t represent the theoretically predicted resistive modulation in the graphene current as noted above. In 2018, Heerema et al. [72] experimentally demonstrated the possibility of DNA sensing with in-plane currents in graphene nanostructures by using a custom-made differential current amplifier that discriminates between the capacitive current signal and the resistive response in graphene. The currents through graphene nanoribbons are relatively large and it is possible to carry out measurements at much higher bandwidths [73,74], which opens up the possibility to sequence DNA information at the translocation speed that is typically observed with solid-state nanopores.

The DNA sequencing can also be accomplished through a graphene nanogap device. The concept is to measure a tunneling conductance across two closely spaced graphene electrodes. The variations of the current can reflect the translocation information of a DNA molecule. In 2010, Postma [75] proposed the idea for the first time. Based on numerical simulation, he demonstrated the single-base resolution of the proposed graphene nanogap sequencing technique. Figure 2c gives the schematic representation of the transverse conductance technique. The individual bases of DNA molecule sequentially occupy a gap in graphene while translocating through it, which is similar to the GNP based technique. Then the Scheicher’s [76] group determined the electrical tunneling current variation at finite bias due to changes in the nucleotides orientation and lateral position by employing DFT and nonequilibrium Green’s function method. The results demonstrate graphene nanogap as a promising approach for rapid whole-genome sequencing applications, although the resulting current was found to fluctuate over several orders of magnitude. Through edge-hydrogenation of the graphene electrodes, the variation in the conductance will be significantly reduced. Meanwhile, the transverse tunneling conductance can be raised by about 3 orders of magnitude, which promises edge-hydrogenated graphene electrode a faster and more reliable technique for DNA sequencing [77]. However, another study proposed that only the G base can be well distinguished from the other three due to the possible occurrence of the quantum interference effects from the rotation of bases and the Fano-type resonances caused by energetic coupling between the discrete energy state of the DNA and the continuous energy states of graphene [78]. The specific tunneling currents of this approach originate from the different chemical composition and structure of nucleobases, which could be influenced by the distance of nanogap, the translocation speed of the DNA, and the interaction between DNA molecule and graphene. There are some researches focusing on the influence factors of this approach and trying to improve the sensitivity, selectivity and efficiency of this device. It was indicated that the best discrimination ability can be achieved with a nanogap distance as 1.1 nm [79], and edge functionalization of hydrogen [77], phosphate-group-grabbing guanidinium ion [80] and nitrogen [81] can be used to reduce the current fluctuation, enhance the DNA recognition sensitivity, and even slow down the translocation speed of DNA. Furthermore, the graphene-hBN heterostructure nanogap was recently proposed as an alternative for DNA sequencing, which could offer high sensitivity [82]. The experiment realization of the DNA sequencing based on tunneling current through graphene nanogaps is challenging. So far, no sequencing test has been conducted though, Postma’s group [83] experimentally studies the translocation of DNA molecules through graphene nanogap, which could open up new advancement for the construction of DNA sequencing devices. In view of the theoretical studies and successes in fabricating tunneling electrodes, interesting experimental data on this area can be expected in near future.

Besides the above approaches, there are also other methods proposed to sequence DNA based on the adsorption of DNA onto graphene sheets. The interaction between graphene and DNA is mainly constituted by π-π bonding, which could be influenced by polarizability of the DNA bases [84,85]. It was reported that the non-covalent adsorption of DNA bases to graphene can induce base-dependent modulations in the current [86,87,88], thus the DNA sequencing can be realized based on this phenomenon. Figure 2d illustrated the stacking of DNA base on a graphene nanodevice during its passage through a fluidic nanochannel. When the DNA strand passes through the nanochannel, the changes in the conductance of the nanoribbon could happen due to the π-π interaction between graphene and nucleobases. The distinct conductance characteristics of graphene allow the different DNA bases to be distinguished. Even though it is extremely challenging to construct such a device because it’s required to make the ribbon narrow enough such that only a single nucleotide is allowed to be adsorbed simultaneously, the ultrasensitivity and low noise features of this technique would attract more and more explorations.

## 3. Pristine Graphene Sheets, GO and rGO Based DNA Detection

Pristine graphene is a 2D carbon sheet with honeycomb-like arrangement of atoms. DNA detection based on adsorption of DNA oligonucleotides onto graphene material was demonstrated with significant potential due to the extraordinary properties, like the high carrier mobility [16] and large surface area [27]. However, the hydrophobic property of pristine graphene makes it hardly be dispersed in water, which limits the applications through DNA adsorption. GO, as a highly chemically modified graphene which contains oxygen functional groups, is broadly used in DNA detection. The oxygen functional groups such as esters, epoxides, hydroxyls and carboxyl lie both on the basal plane and on the periphery [89,90,91]. They can provide pH dependent negative charge and colloidal stability in water and certain organic solvents [89,90,91], which are critical for DNA detection. Though GO has poor electric conductivity, through chemical or physical treatment, the oxygen functional groups on original GO can be partially removed, generating the rGO to restore the electrical conductivity. Depending on the degree of reduction and process, rGO might have different ratio of carbon to oxygen in the final product [92]. These remaining functional groups could offer enhanced interaction with the analyte [93], together with the promising electrical conductivity, the rGO is then proved to be a very attractive material for fabricating the electronic DNA sensors [9]. The uses of graphene-based nanomaterials for DNA detection can be classified into two categories [37]. One is based on charge-biomolecule interactions at π-π domains, electrostatic forces and charge exchange leading to electrical variations in the pristine graphene. The other is to immobilize the molecular receptors onto the surface of GO or rGO through the effect of defects, disorder and chemical functionalization. When the DNA molecules absorb onto the material surface, the interaction between DNA and graphene-based materials could change the electrochemical, electronic, or optical behaviors. Then based on the different signals it can detect, the graphene based DNA biosensors are also generally classified into electrochemical, electronic and optical types, which will be discussed individually.

### 3.1. Electrochemical DNA Biosensors

**Figure 3:**
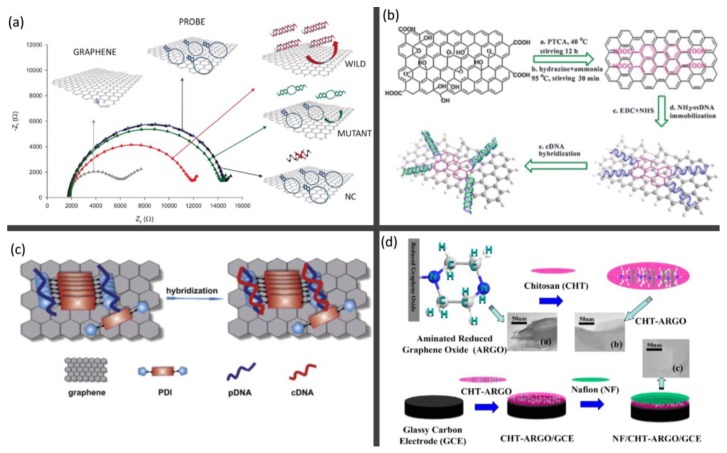
Electrochemical DNA biosensors based on graphene nanomaterials. (**a**) Nyquist plots for the graphene-based sensing platform under pure graphene surface, hpDNA, complementary target and noncomplementary target [96]; (**b**) Schematic representation of graphene functionalization with PTCA, ssDNA immobilization, and hybridization [100]; (**c**) DNA hybridization on PDI/graphene platform [103]; (**d**) The preparation processes of NF/CHT-ARGO/GCE and the corresponding TEM images for each step [113].

Electrochemistry is an important tool for DNA sensing. With the development of many kinds of DNA analysis systems, electrochemical techniques promise the rapid, simple and low-cost detection of DNA and also allow device miniaturization for samples with a very small volume [94,95]. The main principle of electrochemical DNA biosensors is based on the specific hybridization between the probe DNA and the target DNA. The probe DNA is immobilized onto the electrode surface by covalent interaction or physical adsorption to hybridize to the target complementary DNAs (cDNAs). Then the hybridization induces the change in the electrochemical signals, which could be monitored by an electrochemical workstation. Graphene and graphene-based nanomaterials exhibit several advantages, like the large specific surface area and rapid heterogeneous electron transfer, to make it an excellent candidate in electrochemical DNA biosensing. For the pristine graphene platform, the interactions between ring of nucleobases and the hexagonal cells of graphene are dominated by π-π stacking. Partial release of the ssDNA probes from the graphene surface occurs as a consequence of hybridization with complementary target. Based on this concept, Pumera et al. [96] reported the application of graphene platform for hairpin-DNA (hpDNA)-based impedimetric genosensing. As shown in Figure 3a, the charge transfer resistance (Rct) of graphene-modified electrode significantly increased after immobilization of hpDNA onto the sensor surface. After hybridization with the wild-type target, a significant decrease in charge transfer resistance value was observed. The decease of Rct was less significant in the case of hybridization with mutant and no average Rct variation was observed with the noncomplementary (NC) sequence. The reason for the impedance decrease after hybridization with the complementary target was due to the partial release of the hpDNA probes from the electrode surface.

Besides the physical adsorption, a large number of schemes for the immobilization of ssDNA on graphene surface can be used, e.g., covalent immobilization based on graphene’s own oxygenic groups. It was demonstrated that the edge of the GO sheet usually has the -COOH, -OH and -C=O groups, while the basal plane is generally covered with epoxide and -OH groups [97]. Thus, it is generally needed to decorate the basal plane with -COOH for a covalent binding reaction [98]. In 2011, Loh et al. [99] prepared the anodized epitaxial graphene platform with large amount of -COOH groups, and they demonstrated its capability of detecting immobilized DNA and free DNA by covalent grafting or π-π stacking. It indicated that the covalent grafting of probe DNA on anodized graphene affords a larger dynamics range and a more sensitive response than the π-π stacked DNA probe. In order to decorate the graphene sheets with -COOH groups, the methods based on 3,4,9,10-perylene tetracarboxylic acid (PTCA) [100], extremely rapid heating [101] and conjugation of acetic acid moieties [102] were proposed, respectively. The as-functionalized graphene nanosheets were easily dispersed in water and applied for the electrochemical DNA detection. Figure 3b schematically illustrated the procedures of graphene functionalization with PTCA, ssDNA immobilization and hybridization. Due to the π-π stacking and hydrophobic forces [101] between the conjugated rings of PTCA and basal plane of graphene, PTCA could separate the graphene sheets and decorated graphene with plenty of -COOH. Through this functionalized graphene, the signal of negative-charge change and conformation transition upon DNA immobilization and hybridization could be monitored and then the HIV-1 pol gene sequence was satisfactorily detected. To avoid unwanted defects and preserve the integrity of the electronic structure of graphene, various aromatic organic molecules were conjugated to the graphene sheet via π-π stacking interaction. For example, in 2012, Hu et al. [103] proposed using positively charged *N*,*N*-*bis*-(1-aminopropyl-3-propylimidazol salt)-3,4,9,10-perylene tetracarboxylic acid diimide (PDI) to decorate graphene sheets, constructing an efficient DNA impedance biosensing platform. Figure 3c is the schematic representation of DNA hybridization on PDI/graphene platform. The positively charged imidazole rings facilitate the negatively charged ssDNA to be grafted on the graphene surface. The hybridization of cDNA could increase the electrical resistance of the solution and can be detected as the analytical signal for DNA biosensing. Since then, many other aromatic organic molecules, like tryptamine [104], 1-aminopyrene [105], polyaniline [106] and pyrenebutyric acid [107] were successfully anchored to graphene structures through π-π stacking to construct DNA biosensors with high sensitivity and selectivity. Moreover, with large surface-to-volume ratio, improved electrical conductivity and electrochemical activity, rGO is often used to modify the electrode to assist the immobilization of DNA, and enhance the sensitivity and selectivity. The rGO-modified glassy carbon electrode (GCE) was demonstrated as an efficient technique to be applied in the detection of methicillin-resistant staphylococcus aureus DNA [108] and Amelogenin gene [109], and more potential applications can be expected in the future.

The above methods are all based on the hybridization or labeling of ssDNA. The electrochemical DNA label-free DNA detection can also be accomplished through direct oxidation of DNA nucleobases, which could offer the simplest method to detect DNA. In 2009, Zhou et al. [110] developed a chemically reduced graphene oxide modified glassy carbon (CR-GO/GC) electrode for the preparation of electrochemical sensing and biosensing platform. This novel electrode system could overcome the existed drawbacks for most electrode materials, like the relative narrow potential window, high background current and slow electron transfer [111,112]. Then the aminated reduced graphene oxide functional membrane was used to modify the GCE and the device was successfully applied in the detection of guanine and adenine [113]. Figure 3d illustrated the preparation processes of the electrode and the corresponding TEM image of each structure. The chitosan (CHT) is used to improve the electrochemical properties of the membrane and the Nafion (NF) is to enhance the oxidation signals. This functional membrane-modified GCE shows high electro-catalytic properties for the measurement of adenine and guanine. Meanwhile, the rGO based electrochemical DNA biosensors were also synthesized for the detection of DNA damage [114] and the interaction between Cisplatin and DNA [115] based on the oxidation signals. Besides the rGO, other graphene materials, like graphene nanofibers [116], anodized epitaxial graphene [117], and reduced graphene nanowalls [118] were also proposed to prepare the electrochemical label-free DNA detection based on direct oxidation.

### 3.2. Electronic DNA Biosensors

The electronic DNA biosensor, which mainly applies its field-effect characteristics, is generally referred as field effect transistor (FET) based DNA detector. A graphene-based field effect transistor (G-FET) is constituted by a conducting graphene channel across two metal contacts, the source and drain electrodes, through which the current is conveyed. When the G-FET is applied in the DNA detection, the surface of graphene is functionalized with ssDNA probes, which can selectively bind to the target DNA in solution, resulting in an electrical conductivity change associated with biomolecular binding events. Due to the extraordinary properties of graphene, such as large surface area, biocompatibility, ultrahigh mobility, low charge scattering, and ambipolar field effect, G-FETs offer the benefits of high sensitivity, lower detection limits, low cost, and high throughput detection when compared to the existing enzyme-linked immunosorbent assay, Polymerase Chain Reaction, and fluorescence methods [119].

**Figure 4:**
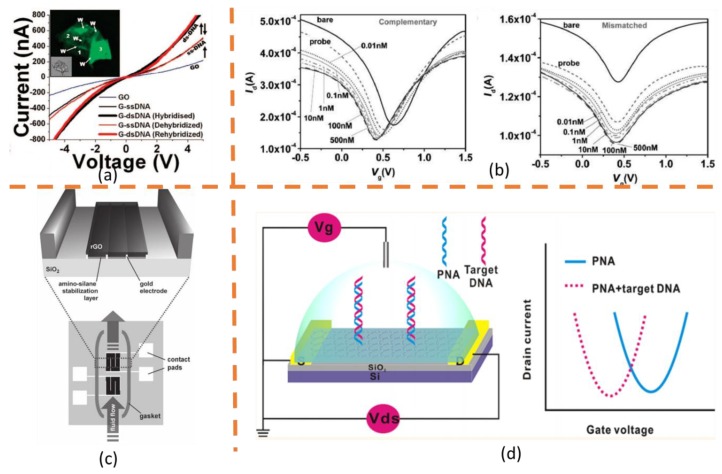
Graphene-based FET DNA detectors. (**a**) DNA transistor: ss-DNA tethering on GO increases the conductivity of the device. Successive hybridization and dehybridization of DNA on the G-DNA device results in completely reversible increase and restoration of conductivity [120]; (**b**) Transfer characteristics for the graphene transistors before adding DNA, after immobilization with probe DNA, after reaction with complementary or one-base mismatched DNA molecules with the concentration ranging from 0.01 to 500 nM [123]; (**c**) Schematic representation of the sensor with a reference part, the enlarged area shows the GO deposited on top of the pre-fabricated electrodes [125]; (**d**) Schematic illustration of the rGO FET biosensor for DNA detection based on PNA-DNA hybridization. The right part shows the typical current signal after the PNA-DNA hybridization [127].

The first attempt of using G-FET to detect DNA was reported by Mohanty et al. in 2008 [120]. They used GO as the semiconductor channel. The target complementary amine modified capturing probe was covalently immobilized onto the surface of GO. Attachment of cDNA could cause a conductivity increase in GO. Moreover, the dehybridization of target DNA using urea was found to be able to almost fully recover the electrical properties of GO, suggesting the robustness and cyclic utilization of this device. Figure 4a shows the confocal images and conductivity test results. After the work of Mohanty, a lot of G-FET based DNA biosensors have been developed using various sensing methods, including back-gated G-FETs [121] and liquid-gated G-FETs [122,123]. It was proved that ssDNAs act as negative-potential gating agents that increase the hole density (n-doping) in single-layer graphene, and there is no significant charge transfer or modification of the graphene band structure in the presence of ssDNA fragments [124]. Due to this electronic n-doping phenomenon, Dong et al. [123] found that the shift in Vg.min (gives the minimum graphene conductance) of the probe DNA immobilized graphene transistors is sensitive to addition of cDNA with a concentration as low as 0.01 nM, which indicated the high sensitivity of their device for the DNA detection. Meanwhile, left shift in Vg.min was less noticeable for the detection of one-base mismatched DNA, further indicated the capability of detecting single-base mutation. Figure 4b gives the results of transfer characteristics with probe DNA, and after reaction with complementary or one-base mismatched DNA molecules, which clearly demonstrated the DNA detection with a concentration as low as 0.01 nM and detection of single-base mutation by using this liquid gating device.

The sensitivity and linear dynamics range of the G-FEA based DNA biosensors is determined by many factors, including graphene quality, surface-to-volume ratio, oxidation degree of graphene, the affinity of probe to graphene, the hybridization efficiency, and the surface coverage of the capture probe. Many techniques were then proposed to enhance the sensitivity and linear dynamics range of the DNA detecting. For example, in 2010, Tamanaha et al. [125] designed a rGO FET device for the real-time DNA detection. They deposited functional groups that preserved active binding sites through the harsh hydrazine reduction process to facilitate the chemical attachment of the biomolecular probe to the rGO. Meanwhile, a reference sensor was incorporated into their platform to mitigate the effects of non-specific biological interactions and improve the specificity. Figure 4c gives the sensor schematic, in which one device act as a reference to eliminate the interference from non-specific biological adhesion. In another study, Xu and his colleagues [122] created a G-FET device for multiplexed electronic DNA array detection. By using CVD graphene, they created a robust array yield which could achieve a sensitivity 10 times higher than the prior state-of-the-art CVD G-FET DNA sensor. And each graphene acts as an electrophoretic electrode for site-specific probe DNA immobilization and performs subsequent site-specific detection of target DNA. In addition, Zheng et al. [126] developed a novel peptide nucleic acid (PNA)-functionalized G-FET biosensor based on CVD graphene which could avoid the contamination and large number of defects in graphene surface. Through a directional transfer technique, they obtained a G-FET DNA biosensor with ultrasensitivity and high specificity. Cai et al. [127] reported a rGO FET DNA biosensor for ultrasensitive label-free detection of DNA using PNA as recognition element, as shown in Figure 4d. PNA is a DNA mimicking molecule that can hybridize with DNA but carries no charge. The uncharged PNA probe enhanced performance of the biosensor by reducing the electrostatic repulsion force between target DNA and capturing probe. By using PNA, the suggested rGO-based FET could achieve a detection limit of 100 fM. The metal nanoparticles can synergistically combine with graphene to achieve better DNA sensing results. It was reported that the Au nanoparticles (AuNPs) could expand the upper detection limit of the G-FET DNA biosensor [123], and Pt nanoparticles (PtNPs) could greatly enhance the detection sensitivity [128]. With the optimized properties, graphene-based biological FETs present great potential in DNA detection.

### 3.3. Optical DNA Biosensors

Graphene materials have outstanding optical properties, which stimulated great interest in the application of optical biosensors. Among the graphene based nanomaterials, GO is mostly researched due to its excellent capabilities for direct wiring with biomolecules, heterogeneous chemical and electronic structure, the possibility to be processed in solution and the tunable electrical properties [129]. The current GO-based optical DNA biosensors are mainly based on the Förster (or fluorescence) resonance energy transfer (FRET) effect, for which GO can actuate as FRET donor or acceptor because it can display both the photoluminescence properties [130,131] and quenching capabilities [132,133]. Also, the GO was reported to be able to fluorescent over a broad range of wavelengths, from near-infrared to ultraviolet [134], and present high fluorescence quenching capabilities, good biocompatibility and large surface areas [135], so GO opens the door to unprecedented optical DNA detection strategies.

**Figure 5:**
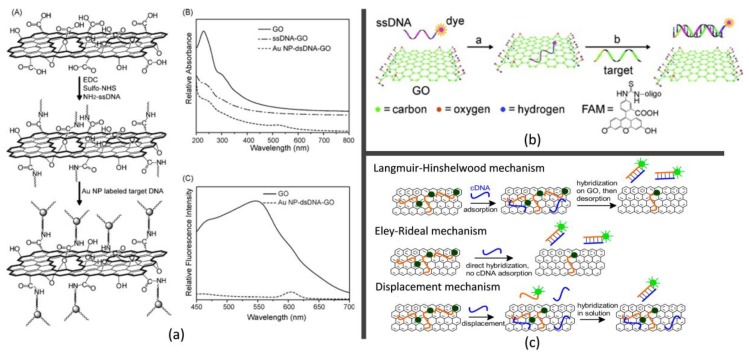
Fluorescence DNA biosensors based on graphene nanomaterials. (**a**) Schematic of the GO-based optical DNA biosensor, the UV-vis adsorption spectra and the relative fluorescence intensities of GO and AuNP-dsDNA-GO [136]; (**b**) Schematic representation of the detection platform when GO was used as acceptor of FRET [138]; (**c**) Three possible mechanisms of hybridization between a probe DNA adsorbed by GO and its cDNA [145].

In 2010, Seo and his coworkers [136] proposed a novel biosensor by employing GO as donor and AuNPs as acceptors of FRET effect for detecting DNA-DNA hybridization interactions. They found the hybridization of AuNPs labeled cDNA with the probe DNA on the GO surface could drastically reduce the fluorescence emission intensity of the GO array, due to the fluorescence energy transfer between AuNPs and the GO sheets. Figure 5a illustrated the schematic of their GO-based DNA biosensor, the UV-vis adsorption spectra and the relative fluorescence intensities of GO and AuNP-dsDNA-GO. It shows that after the probe DNAs on the GO surface are hybridized with AuNP labeled cDNAs, the GO fluorescence emission is reduced by more than 95% owing to the effective quenching by the AuNPs, which suggests the potential of GO as a novel fluorescence label for selective detection of DNA-DNA hybridization. Then Seo [137] systematically studied the FRET efficiency between the photoluminescent GO and Cy3.5 dye by controlling the donor-acceptor distance with a dsDNA and demonstrated that the photoluminescent GO displays a relatively low 21.9% quantum yield and can be used as an acceptor rather than a donor in the FRET pair system. There are many applications by treating GO as FRET acceptor. The first DNA biosensing platform based on GO as acceptor of FRET was reported by Lu et al. [138] in 2009. Figure 5b is the schematic representation of this detection platform. The GO could bind dye-labeled ssDNA and quench the fluorescence of the dye due to noncovalent binding. In the presence of a target, the binding between the dye-labeled DNA and target molecule will alter the conformation of ssDNA and disturb the binding between the dye-labeled ssDNA and graphene. Thus, the dye-labeled DNA was released from the GO, resulting in restoration of dye fluorescence. Based on the quenching properties of GO, both hairpin DNA and molecular beacon (MB) were then designed for homogeneous DNA detection [139,140]. Meanwhile, the same mechanism can be extended to a multiplexed (multicolor) DNA detection [141] or a multiplexed detection of different targets, e.g., DNA, proteins, metal ions, by using MB [142] due to the high planar surface and universal quenching capabilities of GO. The dsDNA can also be detected by the formation of triplex DNA with dye-labeled ssDNA [143] or adsorbing the dye labeled ssDNA onto the GO surface [144]. As revealed by Liu et al. [145], there are three possible mechanisms of hybridization between a probe DNA adsorbed by GO and its cDNA, as shown in Figure 5c. The first is Langmuir-Hinshelwood mechanism, for which the cDNA was also adsorbed followed by diffusion on GO. When the cDNA meets a probe DNA, a duplex is formed on GO. Another possibility is the Eley-Rideal mechanism, where the adsorbed probe DNA directly reacts with its cDNA that is dissolved in the solution phase at the GO/water interface. The third one is called displacement mechanism, where the probe DNA could be displaced by the target cDNA into the solution phase to hybridize with the free cDNA in solution. The displacement mechanism was confirmed by Liu’s work.

There are many factors that can influence the binding of ssDNA to graphene in solution, further influencing the detection efficiency and sensitivity. For example, the carbon-to-oxygen (C/O) ratios in GO might differ at a large level, which could have a great impact on GO’s ability for fluorescence quenching adsorbed dyes, and the binding interactions to ssDNA, resulting in a broad range of DNA detection sensitivity. Results indicated that GO with high C/O ratio bound more strongly to ssDNA and quenched the fluorescence of organic chromophores more effectively than that with low C/O ratio [146]. Also, the binding of ssDNA to graphene in solution may be influenced by ssDNA length, ions, pH, organic solvent and temperature, which could have impact on the hybridization accuracy and efficiency [147]. It was found that shorter ssDNA bound to graphene with higher kinetics and higher adsorption efficiency, and adsorption was also favored by low pH value and high ionic strength.

The GO optical DNA biosensors are generally based on labeling, which could process high sensitivity, good selectivity, etc., while at the mean time, they require the DNA sequences to be previously fluorophore-labeled. Labeling step incorporated into nucleic acid has shortcomings of limited labeling efficiency, complex multistep analysis, and contamination to samples [148]. In order to eliminating the tedious labeling process, different strategies were proposed. Such as the GO-organic dye charge transfer complex fabricated by Loh [149]. Based on ion-exchange strategy, the dsDNA could be distinguished from other biomolecules. Also, by taking advantage of Ag nanoclusters (AgNCs) optical features and GO’s super-quenching capacity, ssDNA-AgNCs were used to detect the target sequence, which could avoid the direct labeling process [150]. Besides, the above mentioned electronic and electrochemical techniques could offer abundant strategies for label-free DNA detection.

## 4. Graphene-Nanoparticle Hybrid Composites Based DNA Detection

Graphene-nanoparticle (G-NP) composites, wherein sheets of graphene, GO or rGO, are decorated with nanoparticles that are a few nanometers to a couple hundred nanometers in diameter [151] are especially alluring because not only do they display the individual properties of the nanoparticles, which can already process beneficial optical, electronic, magnetic, and structural properties that are unavailable in bulk materials, and of graphene, but they also offer a number of highly desirable and markedly advantageous additional unique physicochemical properties and functions in bio-applications in comparison to either material alone [152]. By combining these two excellent and unique modalities as G-NP hybrids, a number of advantageous properties are attained for biosensing applications. For example, graphene can be decorated with metal nanoparticles to achieve a notable increase in electron transfer rate when it is applied on an electrical device, resulting in the significant improved performance of electrochemical biosensors [153,154]. There are a lot studies focusing the applications of G-NPs hybrid composites for DNA detection, which can also be generally divided into three classes: electronic, electrochemical and optical DNA biosensors.

**Figure 6:**
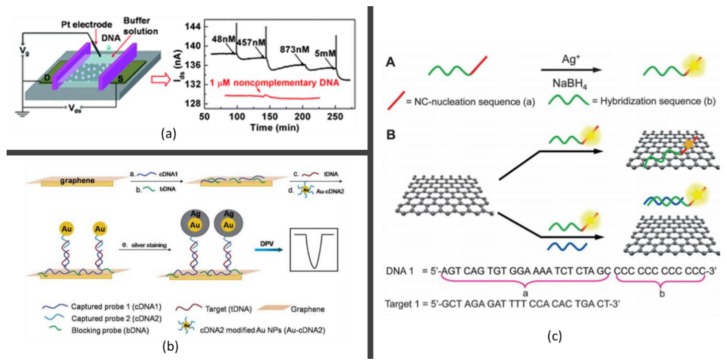
Graphene-nanoparticle hybrid composites based DNA detection. (**a**) Illustration of the solution-based FET device based on PtNPs/rGO films for DNA detection and the typical current signal after the hybridization between target DNA and probe DNA [128]; (**b**) Schematic diagram of the electrochemical DNA sensor based on AuNPs-modified oligonucleotide probe [155]; (**c**) Schematic representation of the preparation of the optical type DNA detection based on AgNCs-GO hybrid materials [150].

In 2012, Yin et al. [128] synthesized a PtNPs/rGO composite and employed it as the conductive channel in a solution-gated FET, which was then used for real-time detection of hybridization of ssDNA. Figure 6a shows the schematic of the device and the real-time recording of the hybridization between target DNA and probe DNA immobilized on PtNPs/GO channels. The adsorption of target DNA due to the hybridization to its complementary probe DNA on PtNPs caused a decrease of current, which indicated its capability in DNA detection. The DNA detection limit of 2.4 nM was reported to be achieved. Recently, AuNPs with different morphology have been designed in DNA-based electrochemical sensors. Lin and her colleagues [155] designed an electrochemical DNA biosensor by the assembly of graphene and DNA-conjugated gold nanoparticles. For this device, the ssDNA was captured on a graphene-modified electrode through the π-π stacking. The target DNA sequence and oligonucleotide probes-labeled AuNPs were able to hybridize in a sandwich assay format, following the AuNPs-catalyzed silver deposition, which could be detected by differential pulse voltammetry. Figure 6b depicts the schematic diagram of this electrochemical biosensor. It was proved that this device has a good analytical performance with a wide detection linear range from 200 pM to 500 nM. Also, it has the capability to discriminate the complementary sequence from the single-base mismatch sequence. On the other hand, Du et al. [156] utilized graphene-mesoporous silica-AuNP hybrids for the ultrasensitive and selective detection of DNA by using strand-displacement DNA polymerization and parallel-motif DNA triplex system as dual amplifications. Their strategy shows high performance for the detection of DNA. Sun et al. [157] presented an electrochemical DNA biosensor for a Listeria monocytogenes assay using an electrochemically reduced graphene/AuNPs nanocomposite modified carbon ionic liquid electrode as the platform. Rasheed and Sandhyarani [158] fabricated an easy DNA biosensor for the ultrasensitive detection of the BRCA1 gene using a “sandwich” detection strategy in which the capture probe (DNA-c) and Au NP-linked reporter probe (DNA-r) DNAs hybridized to the target probe DNA (DNA-t). Wang et al. [159] developed a simple method to fabricate nanoelectrode ensembles (NEEs) by electrodeposition of AuNPs on single-layer graphene oxide sheets coated on a GCE. The fabricated NEEs presented high sensitivity, specificity and stability for DNA detection. Then a super-sandwich electrochemical biosensor based on Au decorated rGO was fabricated by Wang et al. [160] using a methylene blue-labelled signal probe for sequence-specific DNA detection with ultrasensitivity and single-base mismatched target DNA detection. Another novel label-free electrochemical DNA biosensor for rapid detection of multidrug resistance gene based on Au nanoparticles/toluidine blue-GO nanocomposites was proposed by Peng and his coworkers [161]. Under the optimal conditions, the decline of the peak current was linearly related to the logarithm of the concentration of the target DNA from 1.0 × 10^−11^ M to 1.0 × 10^−9^ M, with a detection limit of 2.95 × 10^−12^ M. What’s more, the developed method has the ability to discriminate the MDR1 related DNA sequence from even single-base mismatched DNA sequence. Sun et al. [162] reported an electrochemical DNA biosensor made up of multilayer graphene-AuNPs immobilized with a dual-labelled (50-SH and 30-biotin) stem-loop DNA probe. This DNA biosensor is extremely effective in the detection of the peanut allergen-Ara h1 gene from peanut milk beverages as well as highly sensitive and selective to the target DNA sequence with great recovery (86.8–110.4%). Researchers have also combined GO with noble metal nanoparticles to induce a double-quenching effect that resulted in an increase in the achievable sensitivity. For example, Qu et al. [150] developed an efficient method for the label-free fluorescent detection of DNA based on the exceptional quenching ability of GO and the excellent optical properties of the DNA-silver nanoclusters (DNA-AgNCs). The working principle is schematically represented in Figure 6c. The formation of DNA-AgNCs can be achieved through the use of ssDNA as a template for silver metallization. Upon addition of GO, the fluorescence of DNA-AgNCs can be greatly quenched due to the energy transfer for AgNCs to GO. While in the presence of the target sequence, the binding between the recognition sequence will alter the conformation of the fluorescent DNA probe, which restores the fluorescent intensity. The fluorescent intensity of the probe could provide a quantitative readout of the amount of target DNA. The system presents a simple and costless method to detect DNA with high sensitivity, and also it has the ability for the detection of single-base mismatched sequences. Later, a DNA biosensor based on fluorescence resonance energy transfer between NaYF4:Yb,Er nanoparticle and GO was proposed. The high sensitivity and specificity of this sensor introduces a new method for the detection of DNA with a detection limit of 5 pM [163].

G-NPs hybrid technology for DNA biosensing is very promising even though it is still in its infancy. This technology permits the construction of highly sensitive, selective, customizable, and portable sensors for the detection of variable analytes. It will undoubtedly result in concrete innovations through concerted efforts between multidisciplinary teams which unite chemists, biochemists, material scientists, physicists, biologists and engineers worldwide.

## 5. Conclusions and Prospects

Benefiting from graphene-based nanomaterials’ special properties, many efforts have been directed at developing new DNA biosensors to sequence the nucleic acid base and recognize the specific DNA. In this review, we highlighted the most prominent approaches involving graphene nanopores, nanogaps, nanoribbons, graphene oxide, reduced graphene oxide, and graphene-nanoparticle composites.

For the DNA sequencing, a lot of work was conducted in the nanopore-sensing field based on ionic current detection. Though clear progress has been made, there are still significant challenges existing for this technique, i.e., how to slow down the DNA translocation, how to reduce the stochasticity in the translocation velocity, how to reduce the conformational fluctuations, how to lower the noise levels. The concept of DNA sequencing by monitoring the modulations in the tunneling currents running through graphene nanostructures during interaction with DNA bases has emerged as a more promising method. Therefore, graphene nanogaps and GNRs are synthesized based on the tunneling current detection, which is expected to realize the DNA base discrimination in the near future. Nonetheless, there are few experimental reports on the tunneling current detection due to the limitation of the current experiment techniques. It is anticipated that many more efforts will be put into the experimental realization of the DNA detection based on tunneling current. As for the DNA strand (mainly ssDNA) recognition, three types of biosensors, i.e., electrochemical, electronic, and optical, are focused based on graphene, GO, and rGO nanomaterials. Though the unique properties of graphene, GO and rGO could promise the DNA detection with high selectivity, high sensitivity, and low cost, there are still issues needed to be resolved. For example, the GO fluorescence DNA biosensors are generally based on labeling, which has the shortcomings of limited labeling efficiency, complex multistep analysis, and contamination to samples. The electrochemical DNA biosensors generally need the surface decoration to enhance the immobilization of probe DNA. While the surface decoration could also reduce the electronic transport properties, which will decrease the sensitivity of the detection. What’s more, the FET DNA biosensors are sensitive to the interference factors, such as pH changes and non-specific biological adhesion. To resolve these challenges, many strategies have been proposed. G-NP hybrid structures, which combine the unique and advantageous properties of nanomaterials with those of graphene to produce the advantageous and synergistic effects, are highly expected to improve the performance of the DNA biosensors. Though the G-NP hybrids hold bright future prospects, it is still required to improve the technology to synthesize graphene and its derivatives with controllable size, shape and defects at a low cost and high-yield manner as well as to control the size, composition, morphology and crystallinity of the various incorporated nanoparticles. The formation mechanism of the hybrids and off-site toxicity are also required to be further studied.

Graphene is a special material that offers unexpected opportunities. Though significant challenges are still existing, more fruitful achievements could be expected for the DNA detection based on graphene and graphene-related nanomaterials in the near future. We hope this review could inspire interest from various disciplines and more efforts will be spent for the development of graphene based nanomaterials for bioapplications.
